# Annotations

**Published:** 1894-05-19

**Authors:** 


### ANNOTATIONS.

**Paranoiacs at Chicago.**

An attempt is being made at Chicago to establish a standard of sanity, and if one were not unwilling to incur the charge of levity, it might be said that the *genius loci* is eminently suitable for the task. Chicago is, of course, "the hub of the universe" at the present moment, and the question of the sanity of many of its inhabitants must be one of hourly importance. Dr. W. S. Davis, in the *Chicago Clinical Review*, has published an exceedingly temperate paper, a paper which establishes, beyond controversy, the fact that there is one sane man at least in Chicago itself. There is, contends Dr. Davis, a "borderland," a kind of *regnum protisticum*, in which dwell those persons of whom it is impossible to say that they are insane, and yet which high scientific medical competency would not pronounce to be entirely sane. The persons who dwell in this borderland have been called in England, by Drs. Bucknill and Tuke, "Paranoiacs," and Dr. Davis adopts the appellation with approval. What then are "Paranoiacs?" They are, to use an expressive Americanism, "cranks." They are, as a rule, poorly developed; they are unstable too, and eccentric; often they are vicious as well as whimsical and flighty. The American Dr. Billings, in his dictionary, defines "Paranoia" as "unsoundness of mind, crankiness, insane diathesis, hereditary or chronic mental instability, sometimes used to signify monomania with delusions." The object of Dr. Davis in endeavouring to establish a standard of sanity, is one at which we have often laboured in this journal, namely, the attempting to secure justice in law courts and elsewhere for those members of the human family who are not well endowed with the power of self-protection. A secondary, but important, object is to put an end to the contradictions in expert medical testimony which so frequently disgrace both American and European law courts. Let us establish something like a natural standard of sanity is Dr. Davis' plea; and let both doctors and lawyers make themselves thoroughly intelligent in its comprehension and interpretation. Then we may hope that science and law will see eye to eye; that justice will be done to the feeble-minded, and that medicine will at length be delivered from unmerited public degradation.

**An Unexpected Cure.**

Dr. Cornett, an old gentleman of eighty, has, according to the *Medical News* of Philadelphia, had a very unusual experience, an experience which shows us that even in medicine it is the unexpected which at least sometimes happens. It seems that in consequence of an endocarditis in middle life Dr. Cornett had been subject for twenty years to frequent attacks of excessive rapidity of the cardiac pulsations. The attacks came without warning and disappeared without warning, and their physiology was exceedingly obscure. Dr. Cornett suffered also from bronchitis, and by way of treatment for the bronchitis he took more or less regularly doses of beechwood creosote. What the effect of the creosote on the bronchitis was it is not stated; but the cardiac attacks entirely ceased whilst the remedy was being taken, and for seven months—that is so long as the creosote was continued—they never reappeared. Now, this is very interesting as an isolated fact, but how much more interesting it would be if one could find out why the beechwood creosote cured the palpitations. Was the effect produced upon the cardiac ganglia, or, as is more probable, upon the cardio-inhibitory centre in the brain? At any rate it is clear that beechwood creasote is a substance which has a powerful effect in human physiology, and we would like to know more about it. Let us go a step further. May not the members of all the beech tribe possess active principles which may be of therapeutic service? And if the members of the beech tribe, why not other varieties of forest trees, of shrubs and of plants whose properties have never yet been investigated? We have often professed our conviction that scientific medicine is at the very beginning of her healing power and activity. May we not conclude, from this one little piece of accidentally discovered knowledge of the value of beech creosote, that if the whole vegetable kingdom were systematically, scientifically, and perseveringly interrogated, hundreds of medicinal substances as yet entirely unknown to science would be the rich and welcome reward of the investigation?

**The Lister Portrait.**

Sir Joseph Lister is retiring from active teaching and from hospital work; the more is the pity. His pupils, past and present, are about to do themselves the honour of presenting him with his portrait, and a committee of representative medical men is now in course of formation. Sir Joseph Lister has been an admirable teacher, but he will be remembered as a man of science, whose work marked a distinct advance in the art of medicine of the most profound and far-reaching character, long after the youngest generation of his pupils has passed away. Sir Joseph Lister has combined in his own person the characteristics of a distinguished bacteriologist and of a successful applier of bacteriological science to bedside work. The antiseptic system of medicine and surgery—for it must be remembered that it is medicine as well as surgery which has been converted to asepticism and antisepticism—has conquered new provinces of cure for medicine, has brought in whole regions of darkness and hopelessness to the light of certain knowledge and assured practical success. This it is which makes Listerism the most striking of all the novel features of modern medicine; it is not merely a conquest of knowledge, it is a victory over manifold physical ills which were previously considered impossible of cure. In a short note like the present it is impossible for us to enter upon a detailed and philosophical exposition of Listerism. But such a detailed and philosophic exposition is greatly needed, alike for the profession, which only sees in Listerism an improved surgical method, and for the general public, which sees in it next to nothing at all. That exposition will undoubtedly be forthcoming when medicine shall have become more philosophical and the public shall have learnt to understand medicine philosophically. In the meantime we congratulate the pupils of Sir Joseph Lister on the greatness of their master and on their own appreciation of his rare scientific worth and personal dignity. The movement which is on foot to present Sir Joseph with his portrait cannot fail to evoke sincere and spontaneous enthusiasm.

